# A new ventricular index based on coronal brain magnetic resonance images in patients with idiopathic normal pressure hydrocephalus

**DOI:** 10.55730/1300-0144.5583

**Published:** 2022-12-27

**Authors:** Yunus YILMAZSOY, Serdar ARSLAN, Adnan ÖZDEMİR, Bora KORKMAZER, Osman KIZILKILIÇ, Ali Metin KAFADAR

**Affiliations:** 1 Department of Radiology, Faculty of Medicine, Bolu Abant İzzet Baysal University, Bolu, Turkey; 2 Division of Neuroradiology, Department of Radiology, Faculty of Medicine, İstanbul University-Cerrahpaşa, İstanbul, Turkey; 3 Department of Radiology, Faculty of Medicine, Kırıkkale University, Kırıkkale, Turkey; 4 Department of Neurosurgery, Faculty of Medicine, İstanbul University-Cerrahpaşa, İstanbul, Turkey

**Keywords:** Normal pressure hydrocephalus, index, subarachnoid space, magnetic resonance imaging

## Abstract

**Background/aim:**

The aim of this study was to assess the effectiveness of a new quantitative index for the diagnosis of idiopathic normal pressure hydrocephalus.

**Materials and methods:**

This retrospective study was conducted at İstanbul University Cerrahpaşa Medical Faculty between January 2016 and November 2022. A total of 31 patients diagnosed with idiopathic normal pressure hydrocephalus were included in the study group and 48 patients were included in the control group. Measurement via the new Index was performed on a coronal section of magnetic resonance imaging at the level of the anterior commissure.

**Results:**

The new Index’s mean diagnostic performance was 1.16 ± 0.08 in the study group, significantly lower (p < 0.0001) than the mean of 1.43 ± 0.10 in the control group. When a cutoff value of 1.23 was used for the new index, the sensitivity, specificity, positive predictive value, negative predictive value, and accuracy rates were 96.1%, 90.7%, 80.6%, 98%, and 91.3%, respectively.

**Conclusion:**

The new Index described here is an effective, feasible, reproducible, highly sensitive, and specific quantitative method that can contribute to the improved diagnosis of patients with idiopathic normal pressure hydrocephalus.

## 1. Introduction

Idiopathic normal pressure hydrocephalus (iNPH) manifests in gait disturbance, urinary incontinence, and cognitive dysfunction among patients with ventricular dilation [1]. Although gait disturbance is a major symptom of iNPH, a lack of classical signs in some cases makes rendering an accurate diagnosis difficult. Many clinical, biochemical, and radiological methods are available to help in diagnosis and determine treatment needs [2–4]. A cerebrospinal fluid (CSF) tap test, intermittent CSF lumbar drainage, and CSF outflow resistance measurement are tests which use some of these methods [5, 6]. However, there is no single test considered to be the gold standard for diagnosing the disorder or predicting outcomes if it is treated via surgical shunting.

Radiological assessment may be performed using computed tomography (CT) or magnetic resonance imaging (MRI). INPH can be defined as cases of normal CSF pressure with ventriculomegaly, detected by radiological means [7]. Moreover, a special brain atrophy pattern has been identified in these patients named disproportionately enlarged subarachnoid space hydrocephalus (DESH). This pattern is described as a dilatation of the Sylvian fissures with tight high convexity (narrowed subarachnoid spaces at the vertex) [8–10]. There are publications showing that the DESH finding may be due to focal malabsorption of CSF, suggesting that the expansion in the ventricular system may not be equal in all directions [11]. There is an ongoing struggle to find a reliable and accurate quantitative index for patients with iNPH [12,13].

In the present study, we describe a new quantitative method in coronal MRI images and thus contributing to the diagnosis of iNPH.

## 2. Material and methods

This study was approved by the local ethics committee of İstanbul University- Cerrahpaşa (The approval number: 2022-431441) and retrospectively conducted using patient records, brain MRI images kept between January 2016 and November 2022.

### 2.1. Population

All patients in the study group provided the diagnostic criteria according to the revised Guidelines for iNPH [14]. According to the guidelines, patients were defined as having possible iNPH providing the presence of more than one symptom from the classic iNPH triad (gait disturbance, urinary incontinence, and cognitive impairment), the onset of the symptoms at the age of 60 or older, Evan’s index value is more than 0.3, no presence of underlying diseases or congenital hydrocephalus. Patients with CSF pressure less than 200 mmH_2_O and the tap test or drainage test response or presence of DESH sign with the gait disturbance in addition to possible iNPH criteria were defined as probable iNPH and shunt responders with probable iNPH criteria were defined as distinct iNPH.

**Figure 1:**
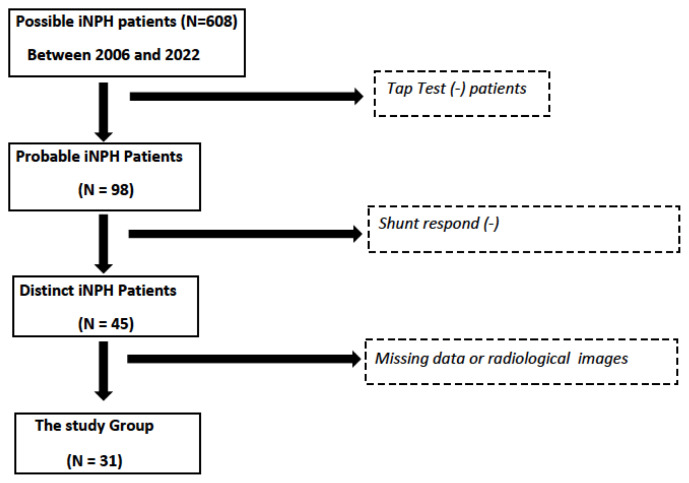
Flowchart of the distinct iNPH patients included in the study group.

There were a total of 31 patients included in the study group, followed by the neurosurgery department, responding to shunt therapy, and meeting the criteria for distinct iNPH. Patients who were diagnosed with possible or probable iNPH were excluded from the study group (Figure 1). Patients with a history of trauma or meningitis, had an underlying disease like brain mass, and had ventricular asymmetry were excluded from the study.

Patients over the age of 60 who were evaluated with a brain MRI because of headache complaints were included in the control group. All patients in the control group were diagnosed with age-related ventriculomegaly without any specific disease detected in brain MRI examinations and clinical evaluations.

A DESH positive scan was determined as the disappearance of sulci on two sequential coronal T1 weighted MRI sections and the presence of tight high convexity, enlarged subarachnoid space at the level of Sylvian fissure, and ventriculomegaly [2, 14].

As per the ventriculomegaly criteria, Evan’s ratio was calculated as the maximum frontal horn width to the maximum inner cranial diameter at the same level [15].

### 2.2. Image analyses

Brain MRIs were performed using a 1.5 Tesla Siemens Magnetom Aera (Siemens Medical Systems, Erlangen, Germany) device, using the standard head coil in a supine position.

The standard brain MR images collected from all participants consisted of axial T2, axial flair, sagittal T2, coronal T2, and coronal T1 sequences. The time to repeat (TR)(ms)/time to echo (TE)(ms)/flip angle/field of view (FOV)(mm)/slice thickness(mm) parameters used were 4700/101/150/220/5 in T2-weighted images, 9000/105/150/220/5 in the flair series and 500/15/90/220/5 in the coronal T1 images. In addition, thin-section axial T2-weighted images (TR: 4.430, TE: 102 ms, slice thickness: 2 mm) were obtained for multiplanar analysis.

### 2.3. Measurement

**Figure 2:**
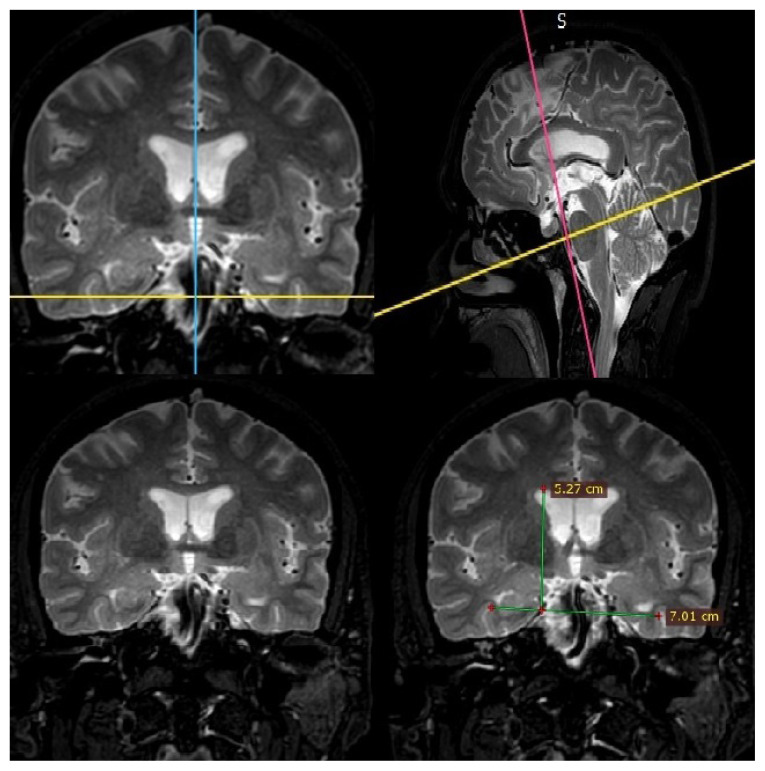
Measurements are performed at the level of anterior commissure on the coronal plane which is set parallel to the brainstem on multiplanar images.

**Figure 3:**
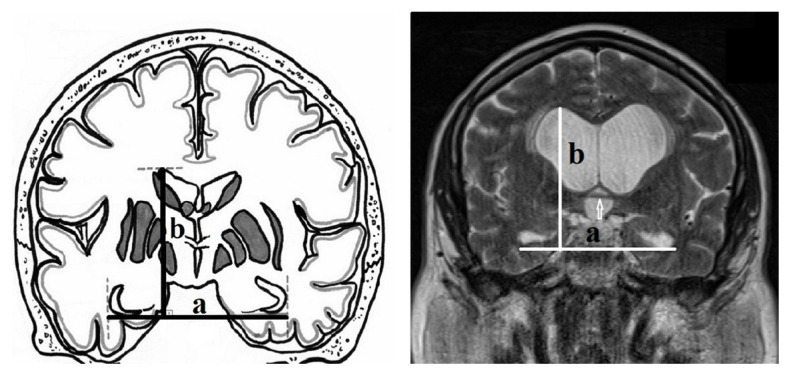
Illustration and T2 weighted MRI image of coronal brain section at the level of the anterior commissure (white arrow) demonstrates the new index, defined as the ratio of the distance between the lateral margins of the temporal horns that pass by the lower margin of the temporal horns (a) and the perpendicular distance between this line and the apex of the lateral ventricle (b).

The new index performed here was calculated based on measurements from T2-weighted multi-planar reconstructed images (Figures 2A, 2B, 2C, and 2D). After adjusting the coronal slices parallel to the brainstem, the measurements were performed at the level of anterior commissure where the lateral wall of the temporal horns of the ventricles was well visualized. This new index was defined as the ratio of the distance between the lateral margins of the temporal horns passing from the lower margins of the temporal horns and the perpendicular distance between this line and the apex of the lateral ventricle (Figures 3A and 3B).

The measurements were carried out by two radiologists (S.A. and Y.Y.) with at least 8 years of experience in their field.

### 2.4. Statistical analyses

All statistical analyses were performed with Statistical Package for the Social Sciences ver.24 (SPSS, IBM corp., Armonk, N.Y., USA) software package. Mean, median, range, frequency, and percentage values were reported in the present study.

Student’s t-test was used to compare mean values. ROC analysis was used to calculate the sensitivity, specificity, cutoff point, and area under the curve. The agreement of the observers was evaluated with the K test. The agreement between the observers was evaluated as poor if the K value ≤ 0.40, medium between 0.41–0.60, good between 0.61–0.80, and very good between 0.81–1.00. Statistical significance was set to p < 0.05.

## 3. Results

In the study group, 14 distinct iNPH patients were excluded because of missing data or preshunt images that could not be accessed. In the control group, 25 patients were excluded from the study because of intracranial mass or hematoma, and 3 patients were excluded due to ventricular asymmetry. There were 31 patients who met the criteria in the study group and 48 patients in the control group. Age, sex distribution, and Evans’ index of the patients are summarized in Table.

**Figure 4:**
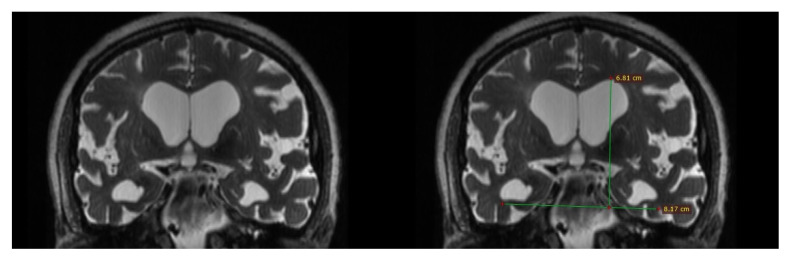
Reconstructed image in coronal plane created from 2 mm thin section T2-weighted images depict enlarged Sylvian fissures in a 74-year-old patient with iNPH. The new index value in this case was calculated as 1.19.

The new index resulted in a calculated mean value of 1.169 ± 0.087 (range 1.03–1.27) in the study group and 1.43 ± 0.09 (range 1.40–1.46) in the control group (Figures 4A and 4B), which were significantly different (p < .0001).

**Figure 5:**
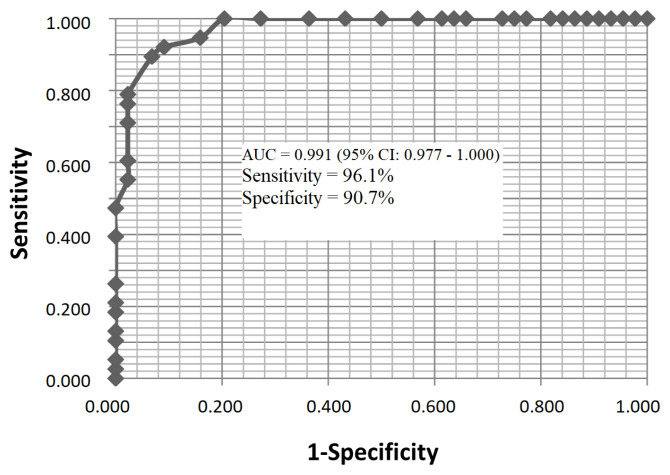
Receiver-operating characteristic curve shows the diagnostic accuracy of the new index.

ROC analysis revealed an area under the curve for the new index of 0.991 (95% CI: 0.977–1.000). The best cutoff value for the new index was calculated to be 1.23. The sensitivity, specificity, positive predictive value, negative predictive value, and accuracy rates were calculated to be 96.1%, 90.7%, 80.6%, 98%, and 91.3%, respectively (Figure 5).

The kappa value was calculated as 0.72, which means there is a good inter-observer correlation.

## 4. Discussion

The diagnostic difficulties associated with iNPH persist and according to the guidelines the definite iNPH can only be diagnosed based on patient response to shunt placement [14]. Despite their diagnostic utility, CSF tap test and CSF lumbar drainage test are invasive methods that can result in hospitalization and increased risk of morbidities [16,17]. Based on these challenges, there is significant justification for the development of new, noninvasive methods for iNPH diagnosis.

Many characteristic features of iNPH are described via diagnostic imaging methods. The Evans index, for example, has been the most popular index of ventricular enlargement used to this end [15]. Despite its lack of disease-specificity, the Evans index can be used to predict ventriculomegaly among patients with normal pressure hydrocephalus. In the present study, all patients had ventriculomegaly and thus an Evans index value of greater than 0.30.

The DESH sign is a qualitative method based on radiological imaging and techniques used for the contribution to the diagnosis and classification of iNPH. Furthermore, the DESH sign has been previously correlated with the shunt response [2,8]. In a study of 83 INPH patients, divided into DESH, incomplete DESH, and non-DESH groups, was used to evaluate the shunt response among these groups [2]. This study’s inclusion of an ‘incomplete DESH’ group is critically important, as the authors also reveal an association in this group with shunt response, indicating that this metric is suited to the often-qualitative nature of a clinical DESH diagnosis where an ‘incomplete DESH’ determination is likely. Although there was a small number of patients in the subgroups, the new index values were not statistically significant between DESH positive and DESH negative groups ​​in our study (p = 0.27).

There is an ongoing search for an effective parameter for use in both the diagnosis of INPH and in predicting the outcomes of its treatment. Benedetto et al. conducted a study on 29 iNPH patients in which they calculated the ratio between the areas of the Sylvian fissure and the subarachnoid space at the vertex (termed the SILVER index). The SILVER index, which is measured based on coronal CT images, had a sensitivity of 82% and a specificity of 96% [13]. In the present study, our newly proposed index has a 96.1% sensitivity and 90.7% specificity. Another index was tested in a study conducted by Yamada et al. on 49 INPH patients (24 tap tests positive and 25 nonresponder participants) and 23 controls. The authors determined that, with quantitative volumetric analyses expansion of the ventricular system in the superior-inferior (z-axis) direction was more prominent than that in the right-left (x-axis) direction. They further defined a novel index (Z Evan’s index), which correlated most robustly with tap test response in INPH patients among those ventricular parameters that were examined [18].

In the present study, the new index measured at the level of anterior commissure indicates a greater enlargement of the ventricular system along the craniocaudal plane (z-axis) than along the horizontal plane (x-axis). The ratio between coronal and axial enlargement of the ventricular system calculated in the same coronal section proved to be beneficial to the sensitivity and specificity of this index. Additionally, we consider that the new index may make an important contribution to efforts to elucidate CSF flow physiopathology among patients with idiopathic normal pressure hydrocephalus.

Iseki et al. revealed that the DESH sign can be seen without ventricular dilatation [9]. Therefore, they thought the DESH sign is caused by disproportionate expansion of subarachnoid spaces, especially at the level of Sylvian fissures, rather than ventriculomegaly. Iseki calculated ventricular expansion by using Evan’s index which is measured in the axial plane. In our study, the new index was defined and measured in the coronal plane. Therefore, these two findings do not contradict each other. We consider the possibility of the dominant ventricular expansion in the coronal plane is primarily compressing the vertex and after then the expansion in the axial plane become visible. In addition, Yamada et al. calculated z-Evan’s index from the level of frontal horns as they described in their study [18]. We believe the expansion of the ventricular system towards the superior especially in the frontal horns will push the brain parenchyma towards the posterior and vertex and thus lead to a tight high convexity appearance that is apparent in the posterior-superior part of the brain. There is a need for further studies to understand the pathophysiologic mechanism.

The retrospective design of the study was its primary limitation. There is also a further need for prospective, larger-scale studies. Another limitation is that routine brain MRI protocols do not contain the thin slice T2 weighted images which decrease the applicability of the new index.

In conclusion, the new index proposed here is an easy-to-use, effective, highly sensitive, and specific quantitative method, which is calculated from coronal MR images and can contribute to the diagnosis of iNPH. We believe it will be of great benefit to future diagnostic studies to further probe the pathophysiology of ventricular enlargement in cases of normal pressure hydrocephalus.

## Figures and Tables

**Table:** Patient characteristics and measurements.

		Study group	Control group	P-Value
Patients (n)		31	48	
Mean age (years)(range)		69.06 (60–78)	71.58 (60–80)	0.124_t_
Male/female		14/17	31/19	0.138_c_
Mean Evan’s index ±SD		0.39 ± 0.09	0.32 ± 0.01	
Mean new index ± SD	Observer 1	1.169 ± 0.087	1.436 ± 0.097	0.0001_t_
	Observer 2	1.177 ± 0.061	1.435 ± 0.095	0.0001_t_
	Kappa value	0.72		

**_t_** student’s t-test; **_c_** chi-square test
